# Comparative proteomics of stenotopic caddisfly *Crunoecia irrorata* identifies acclimation strategies to warming

**DOI:** 10.1111/mec.15225

**Published:** 2019-09-19

**Authors:** Joshua N. Ebner, Danilo Ritz, Stefanie von Fumetti

**Affiliations:** ^1^ Geoecology Research Group Department of Environmental Sciences University of Basel Basel Switzerland; ^2^ Proteomics Core Facility Biozentrum University of Basel Basel Switzerland

**Keywords:** acclimation, climate change, freshwater springs, molecular adaptation, phenotypic plasticity, proteomics

## Abstract

Species' ecological preferences are often deduced from habitat characteristics thought to represent more or less optimal conditions for physiological functioning. Evolution has led to stenotopic and eurytopic species, the former having decreased niche breadths and lower tolerances to environmental variability. Species inhabiting freshwater springs are often described as being stenotopic specialists, adapted to the stable thermal conditions found in these habitats. Whether due to past local adaptation these species have evolved or have lost intra‐generational adaptive mechanisms to cope with increasing thermal variability has, to our knowledge, never been investigated. By studying how the proteome of a stenotopic species changes as a result of increasing temperatures, we investigate if the absence or attenuation of molecular mechanisms is indicative of local adaptation to freshwater springs. An understanding of compensatory mechanisms is especially relevant as spring specialists will experience thermal conditions beyond their physiological limits due to climate change. In this study, the stenotopic species *Crunoecia irrorata* (Trichoptera: Lepidostomatidae, Curtis 1834) was acclimated to 10, 15 and 20°C for 168 hr. We constructed a homology‐based database and via liquid chromatography‐tandem mass spectrometry (LC‐MS/MS)‐based shotgun proteomics identified 1,358 proteins. Differentially abundant proteins and protein norms of reaction revealed candidate proteins and molecular mechanisms facilitating compensatory responses such as trehalose metabolism, tracheal system alteration and heat‐shock protein regulation. A species‐specific understanding of compensatory physiologies challenges the characterization of species as having narrow tolerances to environmental variability if that characterization is based on occurrences and habitat characteristics alone.

## 1 INTRODUCTION

Despite the subterranean location of aquifers, recent studies show that the thermal regimes of groundwater‐fed spring systems are as vulnerable to climate‐induced warming as are other surface waters (Kurylyk, Macquarrie, Linnansaari, Cunjak, & Curry, 2015; Menberg, Blum, Kurylyk, & Bayer, 2014). As a result, stenotopic spring specialists will experience thermal conditions beyond their physiological limits (Duarte et al., 2012; Jyväsjärvi et al., 2015; Sunday et al., 2014). Temperature affects most aspects of organismal biology and ecology, from fundamental macromolecule structures and kinetics of enzymatic reactions to geographical distributions (Gates, 2003; Harrington, Woiwod, & Sparks, 1999; Hochachka & Somero, 2016; Lee, 1991). Populations will have to deal in situ with temperature increase and may undergo adaptive processes through a combination of inter‐generational directional selection acting on pre‐existing genetic variation and intra‐generational phenotypic plasticity, the ability of one genotype to express more than one phenotype when exposed to different environments (Barrett & Schluter, 2008; Chevin, Lande, & Mace, 2010; Fuller et al., 2010; Grenier, Barre, & Litrico, 2016; Hoffmann & Sgró, 2011; Kopp & Tollrian, 2003; Via et al., 1995). The influence of natural selection is extensively studied by quantifying the genetic structure and standing genetic variation for adaptation to changing environmental conditions (Hoffmann & Sgró, 2011). However, selective pressures such as rapid human‐induced climate change might not be mitigated by microevolutionary adaptation on the genetic level alone, leaving environmentally induced plastic responses to the variability of environmental conditions as vital buffer mechanisms of many species (Gienapp, Teplitsky, Alho, Mills, & Merilä, 2008; Lande, 2009; Seebacher, White, & Franklin, 2015). Phenotypic plasticity may then further be under selection and contribute to local adaptation (Harrison, Wright, & Mank, 2012; Levine, Eckert, & Begun, 2011).

Groundwater‐fed spring systems are fragmented, biodiverse ecotones that provide essential ecosystem services (Cantonati, Gerecke, & Bertuzzi, 2006; von Fumetti & Blattner, 2017; Griebler & Avramov, 2014). Climate predictions provide evidence that decreasing precipitation and increasing evapotranspiration will reduce recharge and possibly increase groundwater withdrawal rates (Green et al., 2011; Kløve et al., 2014), reinforcing the likelihood of forced local adaptation due to limiting habitat availability (Fei et al., 2017). Alternatively, to forced local adaptation, range expansion or population extirpation may occur (Walther et al., 2002). Springs in Central Europe have relatively stable environmental conditions, including temperature (Cantonati, Füreder, Gerecke, Jüttner, & Cox, 2012). Spring‐dwelling caddisflies (order Trichoptera) are the most diverse of the strictly aquatic insect orders (Morse, 2011) and function as bioindicators of freshwater quality and habitat integrity (Barquín & Scarsbrook, 2008; De Moor & Ivanov, 2008; Harper, Rosenberg, & Resh, 2006; Hogg & Williams, 1996; Kusch, 2015; Pereira, Cabette, & Juen, 2012). For example, the occurrence of *Crunoecia irrorata* positively correlates with the taxon richness of benthic macroinvertebrates, indicating high conservation value of the springs in which this species is present (Ilmonen, 2008; Rádková, Polášková, Bojková, Syrovátka, & Horsák, 2017; Rychła, Buczyńska, & Szczucińska, 2015). This species is aquatic as larvae and pupae and therefore continually exposed to the prevailing thermal regimes (Mouro, Zatoń, Fernandes, & Waichel, 2016). *C. irrorata* is described as being a spring‐specialist and stenotopic species (Malmqvist & Hoffsten, 2000; Rychła et al., 2015), adapted to the stable thermal conditions found in these systems (Cantonati et al., 2012). These species have by definition, decreased niche breadths and lower tolerances to environmental variability (Lloyd & Gould, 1993 and references therein). Since springs show limited environmental heterogeneity, they are expected to promote diversifying selection in populations, eventually leading to local adaptation (Kawecki & Ebert, 2004). If environments fluctuate temporally, then the number of possible phenotypes that can be expressed from the same genotype should increase (Pfennig et al., 2010). Hence, local adaptation (stenotopicity in the case of spring‐specialists) and phenotypic plasticity can be seen as opposite responses to environmental heterogeneity in space and time (Beldade, Mateus, & Keller, 2011). Freshwater species adapted to thermally stable conditions often evolved a complex of strategies enabling them to thrive at the temperature they experience most often (Angilletta, 2009; Lencioni, 2004). This specialization to thermally stable habitats and the fact that local adaptation occurs despite limited genomic differentiation among populations and high gene flow (Dalziel & Schulte, 2012; Nosil, Funk, & Ortiz‐Barrientos, 2009) suggest the presence of genes involved in local adaptation and potential losses of protein‐coding genes mediating molecular mechanisms to cope with thermal variability (Somero, 2010).

In the present study, we asked if changes in proteome composition of *C. irrorata* reflect past local adaptation, that is if the absence or attenuation of fitness‐increasing molecular mechanisms during acclimation can be quantified and used to infer stenotopy and characterize its hypothesized niche breadth. A proteomics approach allows the study of multiple changes in molecular processes simultaneously (Baer & Millar, 2016; Diz, Martínez‐Fernández, & Rolán‐Alvarez, 2012; Karr, 2008; Silvestre, Gillardin, & Dorts, 2012) and reflects changes at the molecular level close to the organismal phenotype (Wasinger et al., 1995; Wilkins et al., 1996). In the case of strong local adaptation (stenotopicity), we expected little or no fitness‐increasing plastic responses to increasing temperature and multiple responses if the species is eurytopic. Except for the COI gene sequence (Benson et al., 2017), no genomic data for the study species are available. One aim of this study therefore was to opt for a homology‐based approach which would allow the identification of a comparative number of proteins as in nonhomology proteomics studies, utilizing recently published genomes of Trichoptera species. We compared the abundance of hundreds of proteins at three different temperature regimes and characterized molecular mechanisms underlying physiological and plastic responses. By studying molecular adaptation in a species like *C. irrorata*, we provide an initial characterization of the mechanisms used by spring‐dwelling species to mitigate predicted warming of spring water bodies. We further provide new insights into the molecular basis of phenotypic plasticity, discuss the potential and the limitations of this approach and suggest further lines of investigation.

## 2 MATERIALS AND METHODS

### 2.1 Acclimation experiment

*Crunoecia irrorata* larvae were collected from a population at a natural spring in southern Germany (*Herrischried*, 47°39′10″N, 7°58′25″E) in September 2018 and transported to the laboratory within three hours. Organisms were acclimated for 168 hr at 10°C in aerated aquaria. Acclimation and control treatment temperatures were set to 10°C since springs in which *C. irrorata* is commonly found show a water temperature median of 9.4°C (Rychła et al., 2015). After initial acclimation, larvae were placed in test chambers according to Schmidlin, von Fumetti, and Nagel (2015) containing filtered water obtained from the spring. Six biological replicates (BR) were held per treatment at the following temperatures: 10, 15 and 20°C, resulting in 18 BRs. Each BR consisted of three individuals (mean ± *SD*: 240 ± 16 mg). Fifteen degrees can be, if rarely, observed in freshwater springs of Central Europe (Barquín & Death, 2004; Schweiger & Beierkuhnlein, 2014), possibly eliciting a physiological response. Twenty degrees has so far not been recorded in these systems (Von Fumetti, Nagel, Scheifhacken, & Baltes, 2006) and is hypothesized to elicit a significant physiological response of the study organism. To obtain regulated water temperatures (±0.5°C), water was pumped through a continuous‐flow water heater for high temperatures (Hydor T08100 ETH) and a cooling unit for lower temperatures (Aqua Medic Titan 500). Larvae were reared at a light (L)/darkness (D) cycle of L10:D14, and organic material (moss‐covered sticks and leaf litter) was weighed and equally distributed into test chambers. After 168 hr of acclimation to treatment conditions, larval cases were removed and larvae washed with 10× phosphate‐buffered saline (PBS) at room temperature (RT) and subsequently placed on filter paper to remove circumjacent water. Larvae were transferred into ice‐cold 1.5‐ml Protein LoBind tubes (Eppendorf), shock‐frozen in liquid nitrogen and stored at −80°C before protein extraction.

### 2.2 Proteome analysis

Protein extraction and LC‐MS/MS protocols were optimized in previous pilot experiments. After addition of 150 µl lysis buffer (1% sodium deoxycholate [SDC], 10 mM TCEP, 100 mM Tris, pH 8.5 [adjusted with NaOH/HCL]), samples were in‐tube homogenized with a sterile glass pestle followed by 10 min of incubation at −4°C and 10× 1 s ultrasonication (Bandelin Sonopuls HD270). Samples were spun at 5,000 rpm for 10 min at −4°C, and 120 µl supernatant was subsequently transferred to a new tube. Protein concentrations were measured using a Qubit™ 3 fluorometer and a Qubit™ Protein Assay Kit according to manufacturer's instructions. Samples were incubated for 10 min at 95°C at 300 rpm in a Thermomixer C (PCR 96 heating block, Eppendorf). After letting samples cool down at RT, they were spun down at 5,000 rpm for 10 s. Six microlitres of 0.75 M chloroacetamide solution was added to each sample and incubated at 37°C for 10 min at 500 rpm and again spun down at 5,000 rpm for 10 s. After checking if the pH of each sample was between 8 and 9, 1 µl of trypsin (Sequencing Grade Modified Trypsin, Promega) was added to 50 µg extracted proteins per sample which then were digested overnight at 37°C and 300 rpm. Samples were acidified with 80 µl 5% trifluoroacetic acid (TFA) and peptides purified using PreOmics cartridges (Martinsried) according to the manufacturer's instructions. Eluted peptides were transferred to 2‐ml Eppendorf tubes and concentrated to dryness by applying vacuum for 2 hr. Peptides were subsequently dissolved in 20 µl 0.1% formic acid before 1 × 10 s ultrasonication and shaking at 1,4,000 rpm at 25°C for 5 min. After spinning dissolved peptides down at 4,000 rpm for 10 min, peptide concentrations were determined based on absorbance values using a SPECTROstar Nano Absorbance Plate Reader (BMG Labtech). After calculating the final peptide concentrations for each sample, 0.75 µg dissolved peptides was transferred to an LC‐MS/MS vial. Finally, iRT peptides were added to the vial to control for LC‐MS performance, and samples were stored at −20°C prior to LC‐MS/MS analysis.

### 2.3 LC‐MS/MS analysis

Samples were subjected to LC‐MS/MS analysis using an Orbitrap Fusion Lumos Tribrid Mass Spectrometer fitted with an EASY‐nLC 1200 (both Thermo Fisher Scientific) and a custom‐made column heater set to 60°C. Peptides were resolved using an RP‐HPLC column (75 µm × 36 cm) packed in‐house with C18 resin (ReproSil‐Pur C18‐AQ, 1.9 µm resin; Dr. Maisch GmbH) at a flow rate of 0.2 µl/min. The following gradient was used for peptide separation: from 5% B to 12% B over 5 min to 35% B over 65 min to 50% B over 20 min to 95% B over 2 min followed by 18 min at 95% B. Buffer A was 0.1% formic acid in water, and buffer B was 80% acetonitrile, 0.1% formic acid in water.

The mass spectrometer was operated in Data‐Dependent Acquisition (DDA) mode with a cycle time of 3 s between master scans. Each master scan was acquired in the Orbitrap at a resolution of 120,000 full width at half maximum (at 200 m*/z*, MS1) and a scan range from 375 to 1,600 m*/z* followed by MS/MS (MS2) scans of the most intense precursors in the linear ion trap at “Rapid” scan rate with isolation of the quadrupole set to 1.4 *m/z*. Maximum ion injection time was set to 50 ms (MS1) and 35 ms (MS2) with an AGC target of 1.0E6, respectively. Only peptides with charge state 2–5 were included in the analysis. Monoisotopic precursor selection (MIPS) was set to peptide, and the intensity threshold was set to 5.0E3. Peptides were fragmented by HCD (higher‐energy collisional dissociation) with collision energy set to 35%, and one microscan was acquired for each spectrum. The dynamic exclusion duration was set to 30 s.

### 2.4 Bioinformatics and data analysis

#### 2.4.1 Protein Identification

Quality of spectral data (Thermo raw files) was inspected using r v3.5.2 (R Core Team, 2018) and the package rawDiag (Trachsel et al., 2018). Raw spectra were submitted to an andromeda (Cox et al., 2011) search in maxquant (Cox & Mann, 2008) v1.6.3.4. The “match between runs” option was enabled (match time window: 1 min, alignment time window: 20 min). Instrument type was set to Orbitrap, precursor mass tolerance to 15 ppm and fragment ion tolerance to 0.05 Da. Enzyme specificity was set as fully tryptic, with a maximum of two missed cleavages. MS/MS spectra were searched against an in‐house homologous protein database (see below). All searches included a contaminants database (as implemented in maxquant, 267 sequences). For protein identification, unique and razor peptides were used. The peptide spectrum‐match false discovery rate (FDR) and the protein FDR were set to 0.01 (based on target‐decoy approach). Oxidation of methionine (M) and acetylation (Protein N‐term) was specified as variable and carbamidomethylation of cysteines (C) as fixed modifications. Enzyme specificity was set to “Trypsin/P”. Minimum peptide length was seven amino acids.

#### 2.4.2 Construction of a homology‐based sequence database

The database comprised in silico translated gene‐prediction sequences of genomic data of four Trichoptera species: *Stenopsyche tienmushanensis* (Luo, Tang, Frandsen, Stewart, & Zhou, 2018), *Glossosoma conformis* (Weigand et al., 2018), *Sericostoma personatum* (Weigand et al., 2017) and *Limnephilus lunatus* (Poelchau et al., 2015). Predicted protein sequence data were downloaded from respective repositories and fasta files concatenated using command‐line tool cat (Unix). Additionally, to the genomic data, RNA‐seq data of the Trichoptera *Micropterna lateralis* (Hoppeler, Rotter, Krezdorn, & Pauls, 2016) were utilized. The de novo assembled transcript contigs (*n* = 17,759) were downloaded from the specified repository (GenBank, Accession no: GELV00000000). We identified candidate protein‐coding regions using the TransDecoder (TransDecoder.LongOrfs followed by TransDecoder.Predict) pipeline, implemented in the trinity v5.3.0 software distribution (Haas et al., 2013). For the *M. lateralis* transcriptome, 15,243 unique proteins were predicted. Since we were particularly interested in understanding heat‐shock protein regulation in this species, we downloaded heat‐shock protein sequences from the NCBI database (taxonomic scope: Insecta; 6,499 sequences) and concatenated all fasta files as described above. Finally, to remove intra‐database redundancy, we filtered identical protein sequences (*n* = 2,683 removed) using the cd‐hit v4.7 (Fu, Niu, Zhu, Wu, & Li, 2012) clustering algorithm (specified parameters: ‐c 01.00, ‐d 0, ‐t 1). The final database consisted of 83,719 unique sequences. Fasta headers were inspected manually and standardized to forestall complications in the database configuration.

#### 2.4.3 Quantification of protein abundances

Further data analysis was conducted using r v3.5.2 (R Core Team, 2018). Due to comparatively low numbers of commonly identified proteins in all replicates (Figure S2), we calculated pairwise differences in protein abundance of proteins that were present in at least 3 out of 6 biological replicates using the Linear Models for Microarray Data (LIMMA) library (Gentleman et al., 2004; Smyth, 2005) as suggested for few experimental replicates by Schwämmle, León, and Jensen (2013). Protein intensities were calculated through summation of peptide intensities (Carrillo, Yanofsky, Laboissiere, Nadon, & Kearney, 2009) as label‐free quantification (Smaczniak et al., 2012) values showed too many missing values (NAs) for imputation (Figure S5). Intensity values were log_2_‐transformed, and quantile normalization was performed across replicates and conditions via the *normalizeBetweenArrays* function. A linear model was fit for each protein via the function *lmFit* and contrasts from the model fit, and summary statistics were computed via the functions *contrasts.fit* and *eBayes* (Smyth, 2005). We applied stringent log_2_ fold change (FC) cut‐offs of < −2 for lower abundant and >2 for higher abundant proteins. To get an FDR‐based estimate for the set of significant proteins, proteins meeting these thresholds were considered differentially abundant if the adjusted *p*‐value was <.05 (differentially abundant proteins [DAPs]). To identify general protein abundance trends such as increasing or decreasing norms of reaction with increasing temperature (Silvestre et al., 2012), we estimated generalized linear models (GLMs) for continuous response variables using the stan_glm function in the rstanarm R package (Goodrich, Gabry, Ali, & Brilleman, 2018) and subsequently visualized norms of reaction (regression lines and uncertainty estimates) for each protein individually. Each norm of reaction was inspected manually and proteins grouped by increasing or decreasing norms of reaction (examples: Figure S6).

#### 2.4.4 Semiquantitative proteome analysis

In LC‐MS/MS runs, peptides may sometimes not be observed because they are not present; that is, the parent protein is not expressed in a defined experimental group. These quantitatively incomparable proteins are of interest since their treatment‐specific detection may be associated with a true biological effect (Wang, Anderson, Smith, & Dabney, 2012; Webb‐Robertson et al., 2010). To investigate the difference in treatment‐specific identification rate, we analysed every treatment condition individually. The first three majority protein identifiers (Cox & Mann, 2008) corresponded to the set of proteins expressed in any one temperature treatment. In R, protein IDs unique to a temperature were identified using packages *data.table* v1.12.2 (Dowle & Srinivasan, 2019) and *dwtools* v0.8.3.9 (Gorecki, 2015).

#### 2.4.5 Functional annotation of protein sequences

Majority protein ID identifiers of DAPs and proteins unique to a treatment temperature were parsed from the MaxQuant output and corresponding protein sequences extracted from the database using function filter_fasta.py implemented in qiime v1.9.1 (Caporaso et al., 2010). All identified proteins were submitted to a sequence‐similarity search against the *Drosophila melanogaster* reference proteome (ID: UP000000803) using standard stand‐alone blastp v2.2.28+ (specified parameters: blastp ‐outfmt 6 ‐max_target_seqs 2). Assigned UniProt Accession nos were used for GO enrichment analysis using david v.6.8 (Huang, Sherman, & Lempicki, 2009). GO annotation data of all sequences in the default *D. melanogaster* databases were used as background when testing GO term enrichment within the significantly altered proteins (modified Fisher Exact *p*‐value [EASE Score] < .1 for DAPs, increasing/decreasing and secreted proteins [SPs], to allow for discovery sensitivity as suggested by Huang et al., 2009, and conservative Benjamini‐corrected *p*‐value < .05 for temperature‐unique proteins). Secreted proteins (see below) were tested for redundant terms and semantic similarities using revigo (Supek, Bošnjak, Škunca, & Šmuc, 2011) and visualized as treemap graph using R. DAPs were additionally annotated using eggnog v5.0 (Taxonomic scope: Insecta, Orthologs: all orthologs, Gene Ontology evidence: nonelectronic terms, min. hit *e*‐value: 0.001) to obtain distributions of Clusters of Orthologous Groups (COGs) families (Huerta‐Cepas et al., 2019). To estimate the number of SPs in the temperature‐specific proteomes, phobius v.1.01 analyses were used (Käll, Krogh, & Sonnhammer, 2007).

## 3 RESULTS

### 3.1 Differentially abundant proteins

**Figure 1:**
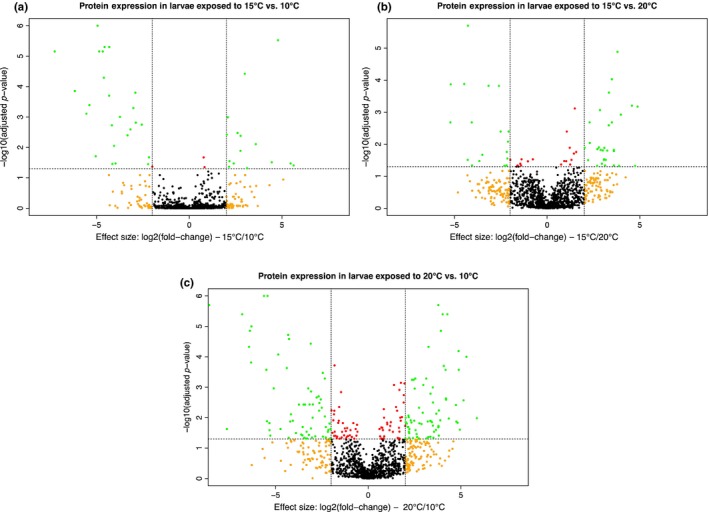
Volcano plots showing (log_2_) fold changes (FCs; *x*‐axis) and (log_10_) adjusted *p*‐values (*y*‐axis) results of proteins commonly identified between temperature treatments. Proteins with statistically significant differential abundance and above or below FC cut‐offs are located in the top right and left quadrants. (a) 15 vs. 10°C. (b) 15 vs. 20°C. (c) 20 vs. 10°C [Colour figure can be viewed at http://wileyonlinelibrary.com]

**Figure 2:**
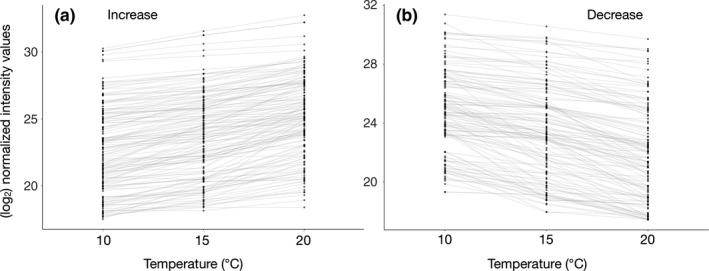
Classification of proteins based on their abundance trends with increasing temperature. Normalized intensity values (log_2_) on *y*‐axis. Mean values of protein intensities identified in up to six biological replicates per temperature are presented as solid dots for each protein. (a) Proteins that show increasing abundance values with increasing temperature. (b) Proteins that show decreasing abundance values with increasing temperature

With the homology‐based approach, 1,358 proteins were identified by peptide data‐dependent LC‐MS/MS, despite the high sample complexity due to the use of whole‐larvae extracts, and 1,212 proteins in at least 3 out of the six biological replicates per treatment. Of these, 37.2% were identified due to the presence of homologous sequences of *M. lateralis*. Sequence data of *G. conformis*, *L. lunatus* and *S. tienmushanensis* contributed between 17.7% and 19.1% each to the overall identification rate. 5.3% of proteins were identified due to the presence of *S. personatum/S. flavicorne* sequences in the search space and 2.5% due to HSP sequences obtained from NCBI (Figure S1). The higher the treatment temperature relative to the control, the more DAPs were identified (Figure 1). Compared to the control, larvae subjected to an increase of 5°C showed 38 DAPs, of which 15 had increased and 23 decreased levels. Fifty‐four proteins were differentially abundant between 20°C and 15°C, of which 34 had increased, and 20 decreased levels in larvae reared at 20°C. A total of 139 DAPs were found when comparing larvae reared at 20°C with the control treatment. Of these, 71 showed increased, and 68 decreased abundance. The full list of annotated DAPs is given in Appendix S2. There were 147 proteins that showed increasing norms of reaction with increasing temperature and 128 proteins with decreasing norms of reaction with increasing temperature (Figure 2).

### 3.2 Protein functional analysis

**Figure 3:**
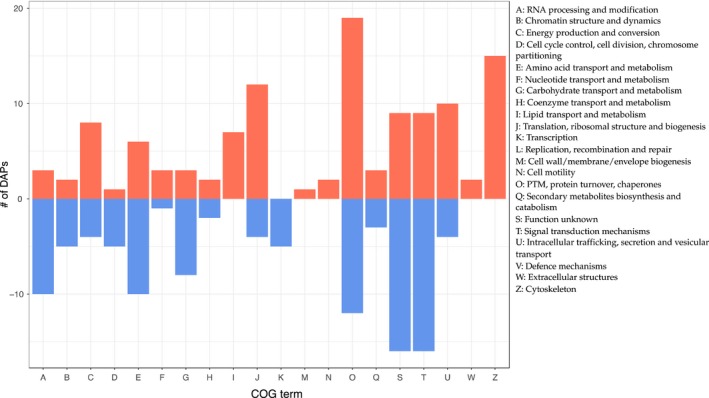
Bar graph showing variability (*y*‐axis) of COG function classes (*x*‐axis) for DAPs. Values below zero show proteins with lower levels at the next higher temperature and values above zero proteins with higher levels at the next higher temperature

To analyse GO distribution within all identified proteins, we performed GO enrichment analysis on the whole data set. Forty‐five GO terms were significantly enriched (Benjamini‐corrected *p*‐value < .05) within all identified proteins, and many were related to functions of proteins usually highly abundant in a cell (e.g., cytoplasmic translation, protein folding, translational initiation and carbohydrate metabolic process; Zhong et al., 2012). Significantly enriched terms for DAPs that were not enriched in the whole data set included chitin‐based embryonic cuticle biosynthetic process, regulation of protein stability, response to stress, regulation of tube size and development of the open tracheal system. Hereby, proteins that increased in abundance were enriched for cuticle‐ and tracheal system‐related GO terms and proteins that decreased in abundance for carbohydrate metabolic process and spermatid development, among others. DAPs were affiliated with a variety of COG terms, including many with unknown function and many that showed increased as well as decreased levels (Figure 3). Proteins with expression regulatory functions such as RNA processing, chromatin structure and transcription showed decreased levels with higher temperatures. Proteins predicted to have functions affiliated with the cytoskeleton, extracellular structures, intracellular trafficking and lipid transport/metabolism showed increased levels with higher temperatures. Proteins showing increasing norms of reaction were enriched for cuticle development, regulation of tube size (open tracheal system), actin filament organization, response to oxidative stress and protein folding. Proteins with decreasing norms of reaction showed similar enriched GO terms as DAPs with decreased levels such as carbohydrate metabolic process and spermatid development.

### 3.3 Semiquantitative proteome comparison and secreted protein analysis

The number of identified proteins varied between treatment conditions when each treatment was analysed separately and more proteins were identified the higher the treatment temperature. In this way 963, 1,157 and 1,446 proteins were identified in 10, 15 and 20°C treatments, respectively (Table 1). 1,075 protein IDs were common to all treatments and when comparing the first three majority protein IDs of each data set (Figure S3).

**Table 1:** Treatment temperatures (*T*, °C), mean peptide concentration, number of identified proteins in proteomes per treatment condition and proteins with N‐terminal secretion signal sequence

Treatment (*T*, °C)	Mean peptide concentration (mg/ml)	Number of identified proteins	Number of SPs (percentage of identified proteins)
10	3.06	963	116 (12.04%)
15	3.49	1,157	116 (10.03%)
20	1.94	1,446	160 (11.07%)

GO enrichment of temperature‐specific protein data sets showed significant (conservative) enrichment of 8 (20 vs. 10°C), 9 (20 vs. 15°C) and 1 (15 vs. 10°C) GO terms such as cytoplasmic translation, mitotic cytokinesis, translational initiation, protein folding and formation of translation preinitiation complex, commonly highly abundant in cells and present within GO enrichment results of the whole data set indicating strong correlation between the whole data set and proteins unique to a temperature. This correlation may be due to the high number of unique proteins between treatments when analysed separately, limiting the explanatory power of this semiquantitative approach.

N‐terminal signal peptide status was predicted for 116 proteins uniquely identified in the 10°C as well as the 15°C treatments and 160 in the 20°C treatment (Table 1). Of these, 66 signal peptides (SPs) were uniquely identified in the 20°C treatment. In a GO enrichment analysis of SPs unique to 20°C, we found enrichment of 22 GO terms, within the following categorical Biological Processes: Chitin metabolic process, retrograde COPI vesicle‐mediated transport, maintenance of epithelial integrity of the open tracheal system/epithelial structure maintenance, regulation of lipid storage and structural constituent of (chitin‐based) cuticle (Figure S3b).

## 4 DISCUSSION

**Figure 4:**
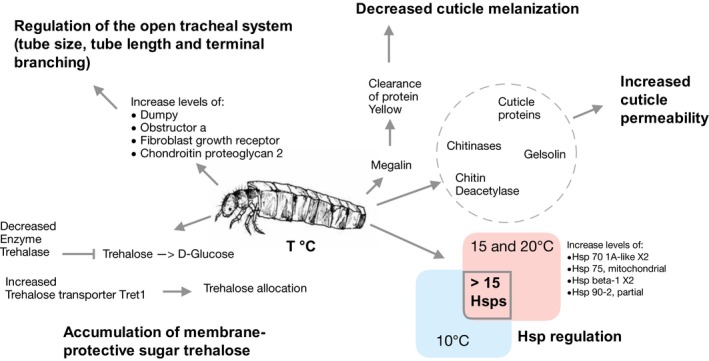
Schematic overview of discussed plastic and physiological responses and candidate proteins playing key roles in these adaptive mechanisms. Major responses include the regulation of trehalose metabolism, hsp abundances, cuticle permeability/melanization and the open tracheal system [Colour figure can be viewed at http://wileyonlinelibrary.com]

Newly synthesized proteins are targeted for entry into secretory pathways by the presence of an N‐terminal signal sequence (Lorey, Rossi, Eklund, Nyman, & Matikainen, 2017; Martoglio & Dobberstein, 1998). These proteins transmit and receive information crucial to the health and adaptive ability of organisms (Walter & Ron, 2011). Of all SPs, 17% were affiliated with the chitin metabolic process or chitin‐based cuticle development. 9% with either chaperone activity or response to hypoxia (such as T‐complex protein 1 subunit alpha and protein disulfide‐isomerase) and 8% had a molecular function related to the plasticity and development of the open tracheal system. Proteins affiliated with these functions were also differentially abundant, showed increasing or decreasing norms of reaction and were unique to noncontrol temperature treatments. As a detailed discussion of all significant proteins and enriched GO terms is beyond the scope of this research article, we focused the discussion on a subset of proteins related to the following responses: altered chitin metabolism and cuticle development, plasticity of the open tracheal system, differential Hsp abundance and accumulation of the membrane‐protective molecule trehalose (Figure 4).

### 4.1 Alteration of cuticle structure

Arthropod cuticles ameliorate perseverance in response to stressors such as predator deterrence (Otte, Schrank, Fröhlich, Arnold, & Laforsch, 2015), thermal acclimation (MacMillan et al., 2016) and other homoeostasis regulators (Sopilniak, Dorfman, & Gruenbaum, 2019). Cuticles consist of proteins, lipids and chitin (*N*‐acetyl‐beta‐d‐glucosamine (GlcNAc); Andersen, Højrup, & Roepstorff, 1995), and environmentally induced phenotypic plasticity of cuticle structures is mediated by the expression patterns of translational products regulating the biosynthesis, degradation or rearrangement of these components: proteins, such as chitin deacetylases and cuticular proteins (CPs), are essential for remodelling chitinous structures (Merzendorfer & Zimoch, 2003; Willis, 2010). The differential abundance of cuticle‐altering proteins hints at two molecular mechanisms mediating acclimation processes: alteration of cuticle permeability and reduction of cuticle pigmentation. Within the proteins unique to the 20°C treatment, multiple insect CPs, larval chitin‐based cuticle development proteins and chitinases were identified as secreted proteins. Chitinases catalyse the hydrolysis of (1‐>4)‐beta linkages of GlcNAc polymers of chitin and are thus essential for cuticle remodelling. Multiple cuticle‐ and chitin‐related proteins such as chitin deacetylase‐like 4, obst‐A and gasp had increased levels at 20°C when compared to the two lower temperatures. Chitin deacetylase‐like 4 also had increased levels at 15°C compared to the control and showed an increasing norm of reaction, together with eight other proteins having cuticle‐related functions. Chitin deacetylases catalyse the reaction by the relationship chitin + H_2_O ⇌ chitosan + acetate (Araki & Ito, 1975). Deacetylation of chitin increases the solubility and decreases the density of chitin fibrils in vitro and may increase cuticle permeability (Geoghegan & Gurr, 2017; Zhao, Park, & Muzzarelli, 2010). Further, the protein gelsolin had increased levels in larvae reared at 20°C when compared to the control. Gelsolin binds both actin‐monomers and filaments, has an actin‐depolymerizing factor domain and can indirectly influence carapace dynamics (Christensen, Owusu, & Jean‐Louis, 2018; Stella, Schauerte, Straub, & Leptin, 1994). Low‐density lipoprotein receptor‐related protein 2 (megalin) was uniquely identified in 15 and 20°C treatments. Megalin promotes endocytic clearance of Yellow, which is deposited into the cuticle before pigmentation and is required for black melanin formation (Kornezos & Chia, 1992; Wittkopp, True, & Carroll, 2002). Due to melanin's efficacy in absorbing solar radiation, megalin detection hints at an involvement of the endocytic Yellow clearance pathway during acclimation. Yellow clearance may limit further melanin biosynthesis and alter light permeability of the cuticle (Riedel, Vorkel, & Eaton, 2010). This phenomenon has been observed in ectothermic organisms, whereby assemblages express comparatively diaphanous cuticles in warm environments (Adamo & Lovett, 2011; Bishop et al., 2016; Fedorka, Lee, & Winterhalter, 2013).

This (semi)quantitatively differential abundance of proteins with cuticle‐altering functions in response to acclimation temperatures occurred within a relatively short time‐scale (<1 week). By reducing insulation and solar radiation absorption, these deduced responses might aid in regulating body temperature and therefore positively shape adaptive capacity. The differential abundances and increasing norm of reaction of chitin deacetylase‐like 4 during acclimation suggest a new biological role for chitin deacetylases in the ecology of insect species.

### 4.2 Respiratory control via the open tracheal system

Warming of water adds to oxygen depletion as it reduces the solubility of oxygen in water and enhances microbial activity (Durance & Ormerod, 2009; Friedrich et al., 2014; Jenny et al., 2016). Adaptations allowing aquatic insects to adjust to more or less severe hypoxic conditions include altering tracheal systems in response to internal PO_2_ allowing for controlled gaseous oxygen exchange (Harrison et al., 2006; Henry, 2004; Hoback & Stanley, 2001). The ability to regulate oxygen delivery during hypoxic conditions and times of increased metabolic demand determines the relative vulnerability of taxa, especially since higher tissue oxygen levels increase oxidative stress (Sohal & Weindruch, 1996). Oxygen limitation shapes thermal tolerances of aquatic tracheates, and respiratory control provides a predictive framework to understand the relative sensitivity of different taxa (Henry, 2004; Jarecki, Johnson, & Krasnow, 1999; Loudon, 1989; Pörtner, 2006; Pörtner & Knust, 2007; Verberk et al., 2016).

DAPs with increased levels at higher temperatures and proteins with increasing norms of reaction were affiliated with the regulation of tube size/length, terminal branching and architecture of the open tracheal system as well as respiratory system development and include, among others, dumpy (isoform M and O), obst‐A and fibroblast growth factor receptor (Muha & Müller, 2013; Tiklová, Tsarouhas, & Samakovlis, 2013; Wilkin et al., 2000). Twenty‐one proteins affiliated with the regulation of the open tracheal system such as sodium‐, potassium‐transporting ATPase subunit beta and chitin‐binding peritrophin‐A are present in acclimation treatment samples but not control (Appendix S2). Further, ten proteins affiliated with cellular response to hypoxia and ROS were unique to the 15°C treatment. These include apolipoprotein D‐like, an evolutionary conserved antistress protein that is induced by oxidative stress (Dassati, Waldner, & Schweigreiter, 2014) and hypoxia up‐regulated protein 1, playing a role in cytoprotective cellular mechanisms triggered by oxygen deprivation (Bando et al., 2017; Ozawa et al., 1999). Nine and 22 proteins affiliated with these respective functions (response to hypoxia/ROS and regulation of the open tracheal system) such as lachesin were identified in larvae reared at 20°C when compared to the control. Lachesin, for example, is required for the maintenance of the trans‐epithelial diffusion barrier (Llimargas, 2003). Proteins with increasing norms of reaction include hypoxia up‐regulated protein 1, apolipoprotein D and neuroglobin, involved in oxygen transport and response to ROS.

These data indicate that respiratory control during increasing environmental temperature is a physiological regulatory mechanism in this species. The ability to regulate gas exchange determines the relative vulnerability of this (and potentially other) freshwater tracheates to the synergistic effects of warming and oxygen limitation and constitutes a phenotypically plastic response since trachea and membrane structure morphologies are altered during the lifespan of the organism. Proteins with ROS defence functions have been shown to be up‐regulated due to increased metabolic activity of ectotherms (Bury, Cichoń, Bauchinger, & Sadowska, 2018), indicating that the observed proteome response to ROS may not be a direct response to hypoxic conditions but to the increased demands on energy metabolism in *C. irrorata* at the experimental temperatures.

### 4.3 Heat‐shock protein response

**Figure 5:**
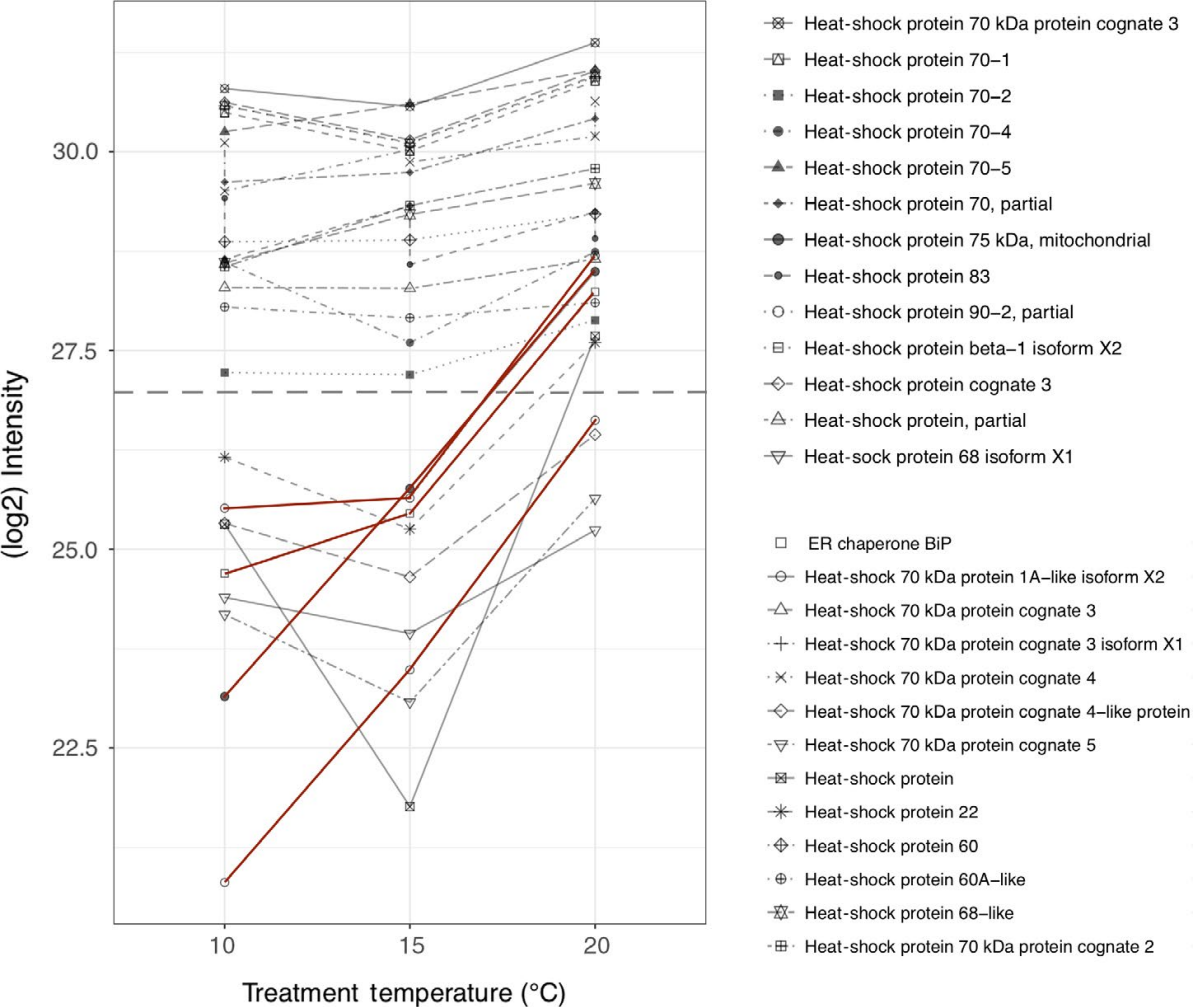
Reaction norms for heat‐shock proteins (hsps) identified in all treatment temperatures. Individual data points are log_2_‐transformed mean intensity values (averaged over biological replicates). Red lines indicate hsps showing increased abundances at higher temperatures. Dotted, grey line separates proteins changing in abundance between temperatures (below) from invariable hsps (above) [Colour figure can be viewed at http://wileyonlinelibrary.com]

A prominent example of a molecular regulatory system is the evolutionary conserved heat‐shock response (HSR) characterized by the expression of heat‐shock proteins (hsp) upon exposure to a variety of physicochemical conditions (Hartl & Hayer‐Hartl, 2002; Hofmann, 2000; Lindquist, 2007; Rosen & Ron, 2002). These chaperones facilitate protein folding and unfolding and play an essential part in both, normal cellular homoeostasis and stress responses by protecting proteins against denaturation and aggregation and therefore set thermal tolerance limits (Johnston & Bennett, 1996; Kregel, 2015; Somero, 2004). Upon exposure of insects to various environmental stressors, synthesis of most proteins declines, but hsp expression usually increases (King & MacRae, 2014). Some species with a local adaptation history to cold environmental conditions show attenuated hsp levels or even lost this essential protective mechanism in response to elevated temperatures (Clark, Fraser, & Peck, 2008; Detrich, Buckley, Doolittle, Jones, & Lockhart, 2012; Oksala et al., 2014). Most hsps identified in all treatments showed no significant norm of reaction variability between temperature treatments, but generally high abundances at all three temperatures could be observed (Figure 5). In the pairwise comparisons, Hsp83 had significantly increased levels at 20 compared to 15°C and hsp22 at 20 compared to 10°C. Hsp83 has previously been shown to increase in abundance after cold hardening in *D. melanogaster* (Qin, Neal, Robertson, Westwood, & Walker, 2005) and *Drosophila auraria* (Yiangou, Tsapogas, Nikolaidis, & Scouras, 1997)*,* postulated to allow ovarian nurse cells to enter a developmental arrest, during heat stress and later resume normal transcription (Lynn Zimmerman, Petri, & Meselson, 1983). Reaction norms (Figure 5) indicate three response curves for hsps: increase (e.g., hsp70 protein 1A‐like and hsp83), U‐shape (e.g., hsp, hsp70 cognate 5 and hsp22) and invariability (e.g., hsp90 and hsp68‐like). Hsps that show constitutive abundance (invariability) between temperatures may facilitate proper protein folding in a cold habitat counteracting the increased protein folding time and the denaturation of proteins at cold temperatures (Dias et al., 2010; Lopez, Darst, & Rossky, 2008; Sørensen, Kristensen, & Loeschcke, 2003; Zwanzig, 2006).

We show that *C. irrorata* has increased levels of certain hsps at higher temperatures, but also that most hsp variants show little variation between low and high temperatures similar to larvae of other species which continually express hsps, likely to protect themselves from cold exposure (Buckley, 2004; Deegenaars & Watson, 1998; La Terza, Papa, Miceli, & Luporini, 2001; Reyes, Corcuera, & Cardemil, 2003). These findings support the notion that the hsp gene family may have been under directional selection in spring‐dwelling species. It might be informative to investigate the effects of thermal acclimation in both, larval and adult stages to compare hsp expression during different life stages as, due to their higher mobility, adult caddisflies are not bound to the habitat and can disperse if environmental conditions become physiologically challenging for the organism. Even though increased hsp levels in the absence of severe short‐term stress hint at the involvement of the hsp gene family in local adaptation to the thermal conditions found in spring systems, it is reasonable to assume that this species is more resilient to long‐term temperature changes as presumed for a stenotopic species, due to the dual roles of chaperones in aiding with the folding of both, cold‐ and heat‐denatured proteins.

### 4.4 Trehalose accumulation and metabolic activity

Trehalose (α‐d‐glucopyranosyl‐α‐d‐glucopyranoside) is a disaccharide constituting the major blood sugar in insects, protecting insect protein and cell plasma membrane integrity during exposure to abiotic stressors such as high temperatures, freezing, high osmosis and dehydration (Elbein, Pan, Pastuszak, & Carroll, 2003; Mitsumasu et al., 2010; Moon, 2002; Shukla, Thorat, Nath, & Gaikwad, 2015). This sugar stabilizes membranes and guards protein degradation by replacing water molecules and facilitating cytosolar vitrification (Benoit, Lopez‐Martinez, Elnitsky, Lee, & Denlinger, 2009; Crowe, Crowe, & Chapman, 1984; Jain & Roy, 2009; Moon, 2002; Singer & Lindquist, 1998; Tanaka et al., 2008).

The enzyme trehalase showed decreased levels in larvae reared at 20°C and the facilitated trehalose transporter Tret1 increased levels at 15 and 20°C when compared to the control. Trehalase is an anomer‐inverting a‐glycoside hydrolase (Lee et al., 2007) that catalyses the hydrolysis of trehalose into two molecules of glucose. Tret1 is a low‐capacity facilitative transporter for trehalose and mediates the bidirectional transfer of trehalose synthesized in the fat body as well as the incorporation of trehalose into other tissue, thereby regulating trehalose levels in the haemolymph (Kanamori et al., 2010).

Similar to the accumulation of sorbitol in whiteflies in response to heat stress (Salvucci, Stecher, & Henneberry, 2000), our data indicate that one testable molecular acclimation mechanism of *C. irrorata* is the accumulation of trehalose, possibly as protection from temperature‐induced cellular damage. The differential abundance of trehalose‐related enzymes and transporters does not necessarily equate the accumulation of trehalose during acclimation, and further experimentation is warranted to confirm trehalose accumulation in *C. irrorata* larval tissue during acclimation to warming.

As body temperatures of ectotherms increase, concomitant increases in both metabolism and respiration can be observed (Neven, 2000; Zuo, Moses, West, Hou, & Brown, 2012). Accordingly, the respiration rate in *C. irrorata* increases with increasing ambient temperature (Figure S4). We hypothesize potentially elevated trehalose levels in *C. irrorata* larvae reared at higher temperatures, and the high demands on metabolic activity create a whole‐organism trade‐off between trehalose accumulation and demands on energy usage. If trehalose accumulation is a fitness‐increasing adaptation in *C. irrorata* to increasing temperatures, a substantial amount of selective fine‐tuning of metabolic regulatory systems, membrane transporter functions and gene regulatory mechanisms has been required to support the use of trehalose as an exaptation (sensu Gould & Vrba, 1982) to environmental conditions, exemplifying that adaptive variation often involves adjusting the production of a type of constituent in ways that enhance fitness.

### 4.5 Potential and limitations of proteomics

Quantitative protein MS has evolved into a versatile suite of methods in molecular ecology research and provides novel opportunities to study and identify molecular mechanisms of adaptation in organisms in response to environmental variability (Baer & Millar, 2016; Diz et al., 2012; Findlay, MacCoss, & Swanson, 2009; Karr, 2008; Biron et al., 2006; Schrimpf et al., 2009). In ecology and evolutionary biology, its regular application is limited mainly due to a lack of established quantitative proteomics methodologies and species‐specific annotated reference protein databases for nonmodel organisms. Despite a large number of identified proteins compared to other thermal acclimation studies, the majority of theoretically present proteins remained unidentified, some of which likely mediate important responses. Based on genome similarities of other Trichoptera species (Table S2), we can assume that the *C. irrorata* genome contains a similar number of protein‐coding sequences as, for example, *L. lunatus* (*n* = ~13,000; NCBI BioProject database, accession PRJNA203303) and that only a fraction of genes are expressed simultaneously (~50%; Bräutigam et al., 2008). The adjusted proteome coverage would then be ~20%, which is not accounting for alternative splicing events. Additionally, dynamic post‐translational modifications (PTMs) are integral to physiological function. They provide mechanisms for activating, changing or suppressing proteins’ functions, but few studies in an ecological context have been conducted (with notable exceptions such as Li et al., 2014). PTMs could be vital to many ecological processes as a way of modulating enzyme activities in response to changes in environmental conditions (Walsh, Garneau‐Tsodikova, & Gatto, 2005), and it would be informative to include PTM analysis in future comparative proteome analyses.

It is also challenging to discern whether observed responses represent unique adaptive mechanisms of the studied species or whether they are shared between species of the same order or even taxon (“phylogenetic baggage”), making it challenging to identify species‐specific adaptations and responses unless comparative physiological studies investigating inter‐specific proteomes are conducted.

Even though species‐specific genomic information would be ideal in any proteomics study, we demonstrate that a homology‐based LC‐MS/MS shotgun proteomics approach is a promising tool for ecological and evolutionary research into the nature of molecular adaptations of nonmodel species. The ability to identify multiple physiological and plastic responses to altered environmental variables in one experiment enables comprehensive studies of adaptive capacity in virtually any species, especially since genome sequences of nonmodel organisms are accumulating at an unprecedented rate (Ellegren, 2014).

### 4.6 Local adaptation to springs

We question the characterization of *C. irrorata* as a stenotopic species based solely on deducing local adaptation from the occurrence in a thermally more or less stable habitat. Differentially abundant proteins and protein reaction norms revealed a clear plastic response for several biological processes within the population in response to temperature changes. The ability to tolerate or adapt to only a small range of environmental conditions (i.e., reduced plasticity (Meier et al., 2014)) does not fit with the presence of the here identified fitness‐increasing physiological and plastic responses when exposed to increasing temperatures and the wide dispersion of the species (Bergan, 2015), indicating that *C. irrorata's* postacclimation resilience to thermal variability, together with the environmental variability of their spring habitats, may be higher than previously assumed. This species‐specific understanding of molecular mechanisms guiding adaptive responses refines our understanding of the vulnerability of freshwater ectotherms to warming and complements data on marine and terrestrial ectotherms (e.g., Pinsky, Eikeset, McCauley, Payne, & Sunday, 2019). Our findings further complement data indicating that species from cold and thermally stable environments are less sensitive to temperature variation than species from warm and variable environments (Seebacher et al., 2015) and that aquatic organisms show high thermal plasticity (Gunderson & Stillman, 2015). These processes may facilitate colonization of other habitats that have higher thermal variability since acclimation shifts performance towards it's optimum and may be beneficial by stabilizing reaction rates across temperature gradients (Seebacher et al., 2010; St‐Pierre, Charest, & Guderley, 1998). On the other hand, plastic and physiological adjustment mechanisms may be energetically too costly in the long term (Seebacher, Davison, Lowe, & Franklin, 2005) and may fail to provide the means for populations to continually track changing climatic conditions, for which evolutionary responses will be required, calling for integrative multi‐omics approaches to disentangle intra‐ and inter‐generational local adaptation to changing environmental conditions.

## AUTHOR'S CONTRIBUTIONS

The experiment was designed and field work was conducted by S.F. and J.N.E. The experiment, molecular work, bioinformatics analyses were conducted and the data were analysed by J.N.E. Mass spectrometry work was conducted by D.R. The manuscript was written by J.N.E. All authors edited the final manuscript.

## Data Availability

The mass spectrometry proteomics data have been deposited to the ProteomeXchange Consortium via the PRIDE partner repository (Perez‐Riverol et al., 2019) with the data set identifier PXD014052. Raw intensity values, results from LIMMA, GO enrichment and Phobius analyses and protein annotations are given in Appendix S2 (combining quantitative with annotation data). The homology protein database, the commands used to construct the database and the data‐DOI(s) specifying where the original genomic data of the Trichoptera species are deposited are available via the DRYAD data depository https://doi.org/10.5061/dryad.r3t28r6.
