# Supplementary material for: Comparative proteomics of stenotopic caddisfly Crunoecia irrorata identifies acclimation strategies to warming
Source: Mol Ecol. 2019 Sep 19;28(19):4453–69. doi: 10.1111/mec.15225 (PMC6856850; doi:10.1111/mec.15225)
Supplement: Supplementary file 1 [file MEC-28-4453-s001.pdf]

MOLECULAR ECOLOGY

Supplemental Information for:

Comparative proteomics of stenotopic caddisfly *Crunoecia irrorata* identifies acclimation strategies to warming

Joshua Niklas Ebner, Danilo Ritz, Stefanie von Fumetti

Contents:

Supplemental tables

Table S1 Peptide concentration measurements of biological replicates at the three treatment temperatures (10, 15, 20 °C).

Table S2 Correlation-table of *in silico* identity extent of pairwise comparisons of genomes of the four Trichoptera species used in this study to generate the homology-based protein database for protein identification.

Supplemental figures

Figure 1 Pie chart showing the distribution of identified protein sequences in reference to their origin in the *in-silico* digested sequence in the homology-based AA-sequence database

Figure 2 Barplot showing the number of identified proteins and in how many of the 18 samples they have been identified

Figure 3 Venn diagram showing the first three majority protein IDs similar and unique to a treatment temperature and results of REViGO semantic analysis of over-represented GO terms (Biological Process) associated with secreted proteins in 20 °C treatment

Figure 4 Dotplot showing data of oxygen consumption rates of *C. irrorata* larvae at temperatures 20 and 30 °C

Figure 5 Plot showing the number of missing values (NAs) of label-free quantification (LFQ) values in the MaxQuant output

Figure 6 Example norms of reaction plots for heat shock protein 22 (increasing) and translation factor waclaw, mitochondrial (decreasing)

References

SI1 Table 1. Peptide concentration measurements of each biological replicate at the three treatment temperatures (10, 15 and 20 °C) using a Spectrostar Nano plate reader (BMG Labtech)

Well Row	Well Col	Content	Raw Data (2601)	Raw Data (2802)	Raw Data (3403)	Baseline Correction 340	Concentration (1.1OD=1mg/mL) in mg/mL (Concentration (1.1OD=1mg/mL))	Volume Sample for 20ul at 0.5 ug/ul	Volume LC-buffer to fill	Temperature (°C) and replicate
**A**	10	Blank B	7.081	7.141	7.189	-0.047	-0.04	-250	270	Blank
**A**	11	Blank B	0.892	0.802	0.693	0.109	0.1	100	-80	Blank
**B**	10	Sample X1	21.432	19.925	11.826	8.1	7.36	1.358696	18.6413	10_1
**B**	11	Sample X2	3.713	3.486	2.327	1.159	1.05	9.52381	10.47619	10_2
**C**	10	Sample X3	9.248	8.697	4.137	4.56	4.15	2.409639	17.59036	10_3
**C**	11	Sample X4	6.105	5.799	3.701	2.098	1.91	5.235602	14.7644	10_4
**D**	10	Sample X5	5.799	5.597	3.473	2.123	1.93	5.181347	14.81865	10_5
**D**	11	Sample X6	5.868	5.746	3.591	2.155	1.96	5.102041	14.89796	10_6
**E**	10	Sample X7	3.232	3.227	1.809	1.419	1.29	7.751938	12.24806	15_1
**E**	11	Sample X8	14.862	13.788	7.949	5.839	5.31	1.883239	18.11676	15_2
**F**	10	Sample X9	3.715	3.48	2.121	1.36	1.24	8.064516	11.93548	15_3
**F**	11	Sample X10	9.354	8.622	4.633	3.989	3.63	2.754821	17.24518	15_4
**G**	10	Sample X11	19.972	18.44	10.62	7.82	7.11	1.40647	18.59353	15_5
**G**	11	Sample X12	5.613	5.343	2.69	2.653	2.41	4.149378	15.85062	15_6
**H**	10	Sample X13	5.656	5.224	2.711	2.513	2.28	4.385965	15.61404	20_1
**H**	11	Sample X14	2.599	2.465	1.314	1.151	1.05	9.52381	10.47619	20_2
**B**	10	Sample X1	10.409	9.504	6.682	2.822	2.56	3.90625	16.09375	20_3
**B**	11	Sample X2	9.287	8.51	5.648	2.862	2.6	3.846154	16.15385	20_4

**C**	10	Sample X3	9.727	9.088	6.724	2.363	2.15	4.651163	15.34884	20_5
**C**	11	Sample X4	2.119	2.11	1.043	1.067	0.97	10.30928	9.690722	20_6
**A**	10	Blank B	0.802	0.755	0.665	0.09	0.08	125	-105	Blank
**A**	11	Blank B	0.84	0.763	0.655	0.108	0.1	100	-80	Blank
**B**	10	Sample X1	10.409	9.504	6.682	2.822	2.56	3.90625	16.09375	20_3
**B**	11	Sample X2	9.287	8.51	5.648	2.862	2.6	3.846154	16.15385	20_4
**C**	10	Sample X3	9.727	9.088	6.724	2.363	2.15	4.651163	15.34884	20_5
**C**	11	Sample X4	2.119	2.11	1.043	1.067	0.97	10.30928	9.690722	20_6

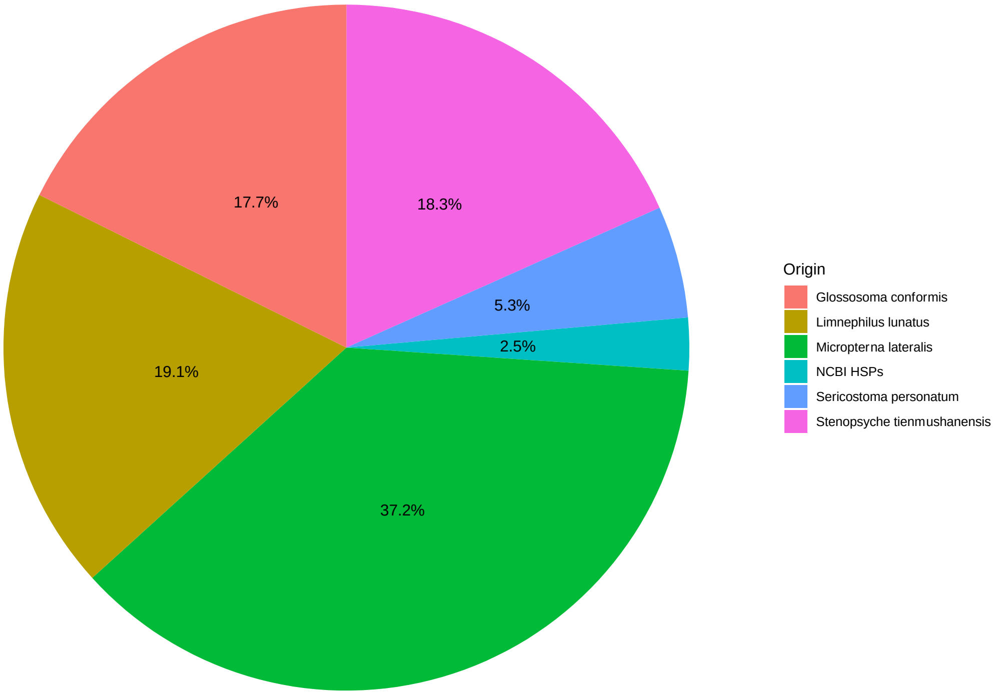

SI1 Figure 1. Distribution of identified protein sequences in reference to their origin in the *in-silico* digested sequence in the homology-based AA-sequence database.

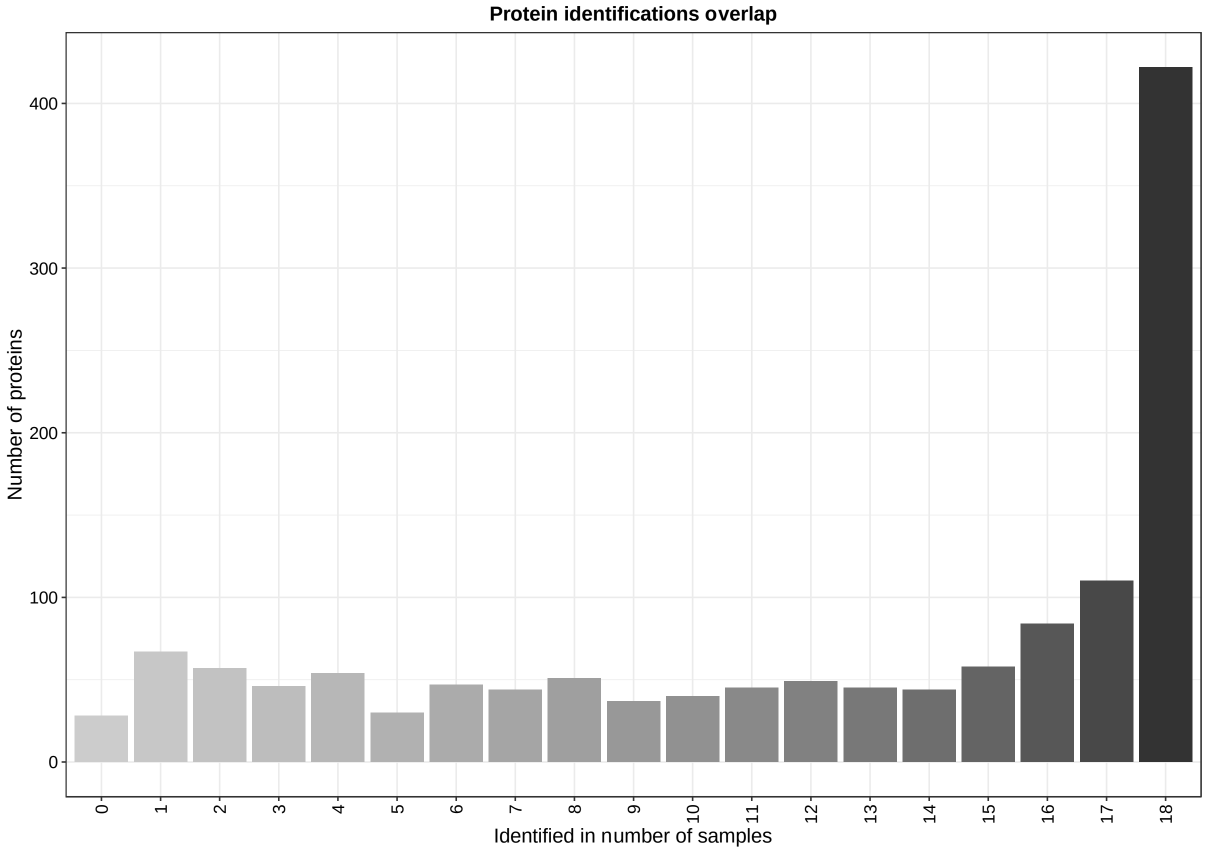

SI1 Figure 2: Barplot showing the number of proteins (y-axis) and in how many of the 18 samples they have been identified. The dataset contains proteins which were quantified in all replicates (bar 18). Some proteins were only identified in a single biological replicate (bar 1). Plot was generated in R using function plot_frequency from package DEG (Zhang et al., 2018).

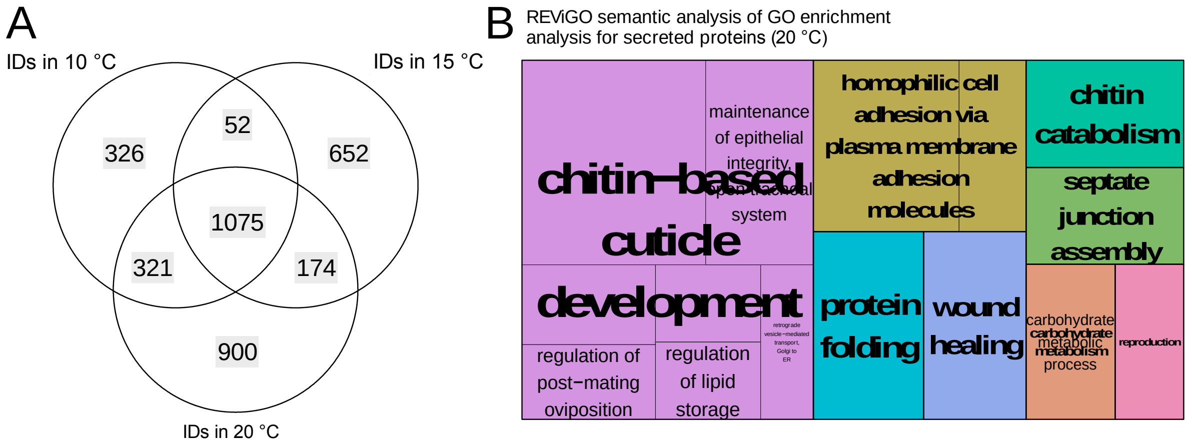

SI1 Figure 3. (A): Venn diagram showing the first three majority protein IDs similar and unique to a treatment temperature. (B): Results of REViGO semantic analysis of over-represented GO terms (Biological Process) associated with secreted proteins in 20 °C treatment. All terms are included with a P-value cut-off at 0.05 from the enrichment analysis (DAVID). Colors mark semantic similarity, whereas the size of the corresponding area reflects the P-value of DAVID enrichment.

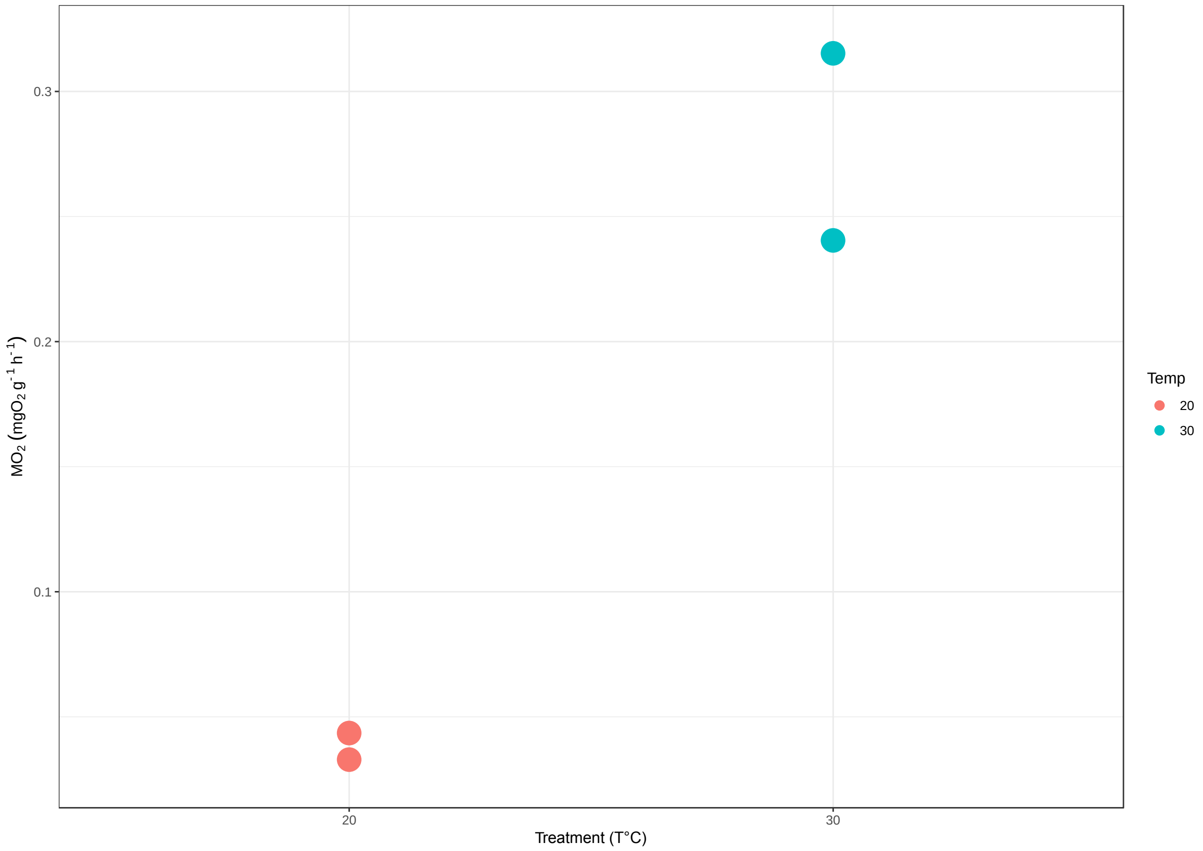

SI1 Figure 4: Dotplot showing data of oxygen consumption rates (y-axis) of *C. irrorata* larvae at temperatures 20 and 30 °C (x-axis; 2 replicates each). Oxygen concentration in the headspace of a closed respirometer (glass vial), connected to a FireSting O_2 fiber-optic oxygen and temperature meter (PyroScience) was measured over 5 hours. Oxygen consumption rate (mg O_2 per gram of animal per minute) was calculated according to formulae (1) of (Steffensen, 1989), using time course of oxygen concentration (slope of regression line) volume of water in respirometer (ml), wet weight of individual (mg) and time period over which measurements were taken as input parameters.

SI1 Table 2. Correlation-table of *in silico* identity extent of pairwise comparisons of genomes of the four Trichoptera species used in this study to generate the homology-based protein database for protein identification. Average Nucleotide Identity scores (ANIm) based on function "dnadiff" implemented in MUMmer (Kurtz et al., 2004) and Tetra (Teeling, Meyerdierks, Bauer, Amann, & Glöckner, 2004) scores (in parentheses) are shown. Calculations were performed with JSpeciesWS (Richter, Rosselló-Móra, Oliver Glöckner, & Peplies, 2016).

	S. tienmushanensis	*S. personatum*	*L. lunatus*	*G. conformis*
S. tienmushanensis	*	85.71 (0.07)	84.81 (0.30)	84.31 (0.64)
S. personatum	85.74 (0.18)	*	84.24 (0.54)	86.08 (0.46)
L. lunatus	84.79 (0.41)	84.24 (0.40)	*	85.57 (1.55)
G. conformis	84.31 (0.73)	86.04 (0.29)	85.51 (1.07)	*

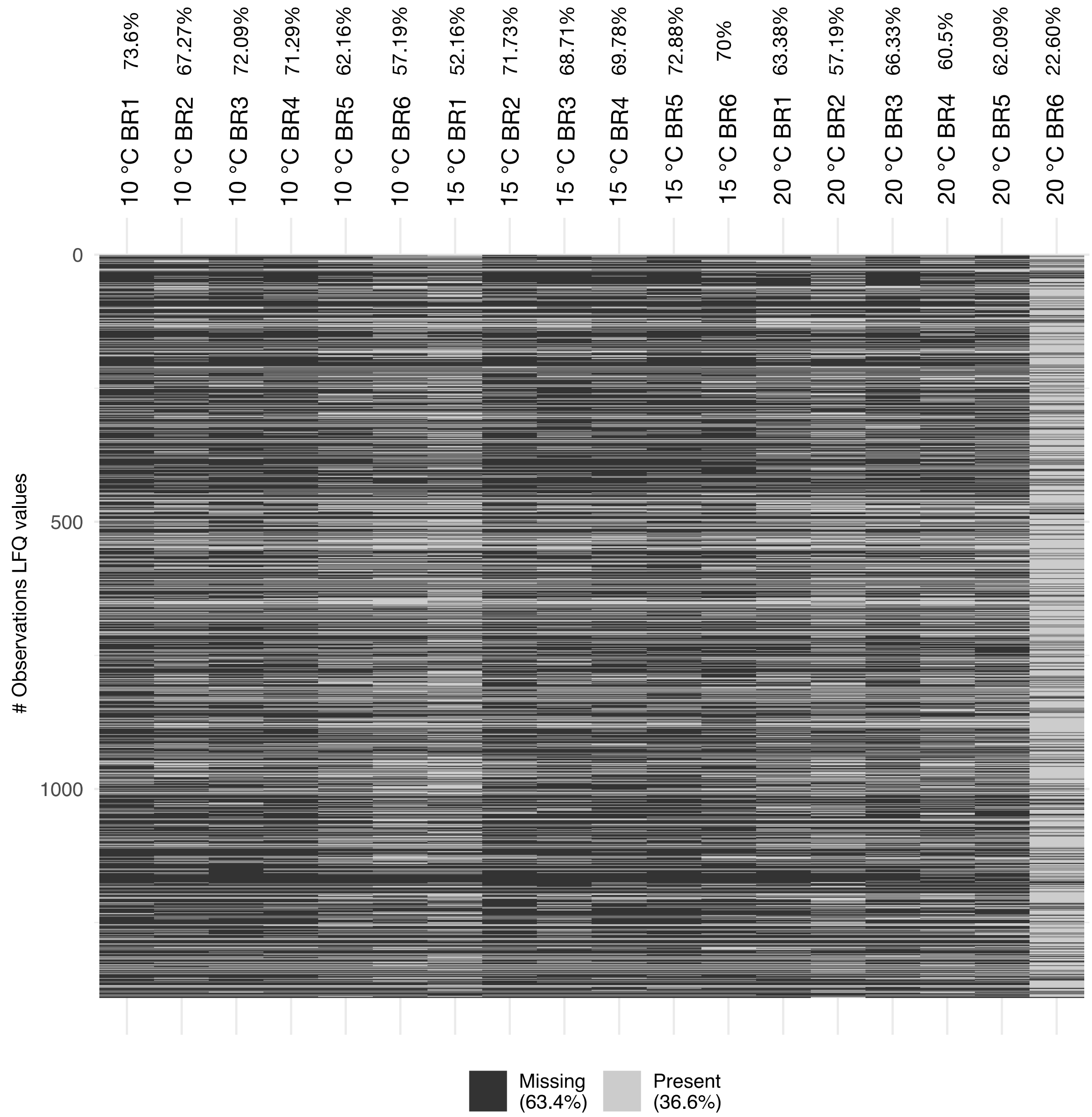

SI Fig. 5: Number of of missing values (NAs) of label-free quantification (LFQ) values in the MaxQuant output. Plot was generated using the vis_miss function of package visdat (Tierney, 2019).

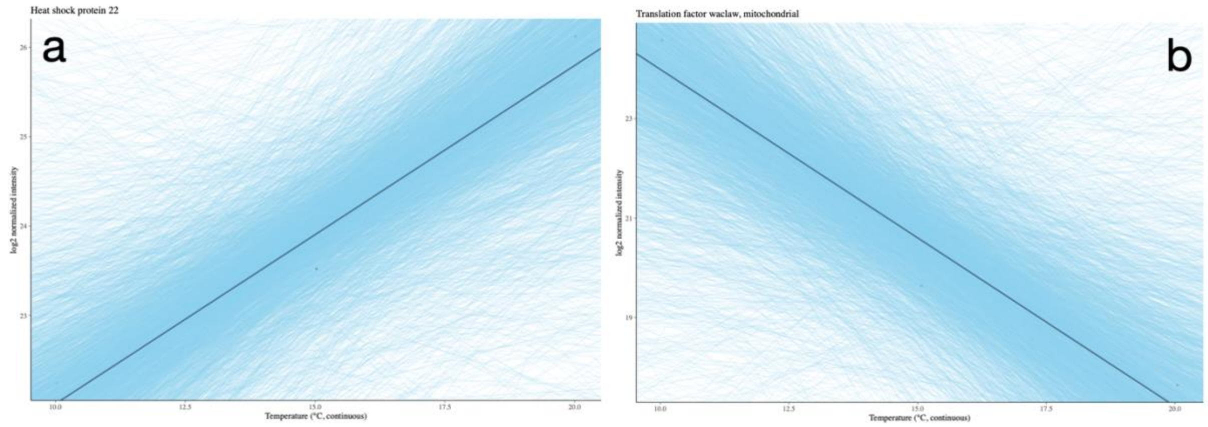

SI1 Fig. 6: Example norms of reaction for heat shock protein 22 (increasing) and translation factor waclaw, mitochondrial (decreasing). Normalized, log2-transformed intensity values on y-axis, treatment temperature (°C, as continuous variable) on x-axis. Plots were generated only for proteins identified at all three temperatures. **a**) Increasing norm of reaction **b**) Decreasing norm of reaction. Regressions model predicting intensity values of individual proteins given their abundances at the three treatment temperatures were computed with the function stan_glm from the rstanarm R package (Goodrich, Gabry, Ali, & Brilleman, 2018). Results from the model fit such as regression lines and estimates of uncertainty were plotted using the ggplot2 R package (Valero-Mora, 2015).

References for Supporting Information 1

Goodrich, B., Gabry, J., Ali, I., & Brilleman, S. (2018). rstanarm: Bayesian applied regression modeling via Stan. R package version 2.17.4. Comprehensive R Archive Network (CRAN). Retrieved from https://cran.r-project.org/web/packages/rstanarm/index.html

Kurtz, S., Phillippy, A., Delcher, A. L., Smoot, M., Shumway, M., Antonescu, C., & Salzberg, S. L. (2004). *Open Access Versatile and open software for comparing large genomes* (Vol. 5). Retrieved from http://www.tigr.org/software/mummer.

Richter, M., Rosselló-Móra, R., Oliver Glöckner, F., & Peplies, J. (2016). JSpeciesWS: A web server for prokaryotic species circumscription based on pairwise genome comparison. *Bioinformatics*, *32*(6), 929–931. doi:10.1093/bioinformatics/btv681

Steffensen, J. F. (1989). Some errors in respirometry of aquatic breathers: How to avoid and correct for them. *Fish Physiology and Biochemistry*, *6*(1), 49–59. doi:10.1007/BF02995809

Teeling, H., Meyerdierks, A., Bauer, M., Amann, R., & Glöckner, F. O. (2004). Application of tetranucleotide frequencies for the assignment of genomic fragments. *Environmental Microbiology*, *6*(9), 938–947. doi:10.1111/j.1462-2920.2004.00624.x

Tierney, N. (n.d.). Preliminary Visualisation of Data [R package visdat version 0.5.3]. Retrieved from https://cran.r-project.org/web/packages/visdat/index.html

Valero-Mora, P. M. (2015). ggplot2: Elegant Graphics for Data Analysis. *Journal of Statistical Software*, *35*(Book Review 1). doi:10.18637/jss.v035.b01

Zhang, X., Smits, A. H., Van Tilburg, G. B. A., Ovaa, H., Huber, W., & Vermeulen, M. (2018). Proteome-wide identification of ubiquitin interactions using UbIA-MS. *Nature Protocols*, *13*(3), 530–550. doi:10.1038/nprot.2017.147
